# Multifunctional CuBiS_2_ Nanoparticles for Computed Tomography Guided Photothermal Therapy in Preventing Arterial Restenosis After Endovascular Treatment

**DOI:** 10.3389/fbioe.2020.585631

**Published:** 2020-10-21

**Authors:** Xiaoyu Wu, Kun Liu, Ruihua Wang, Guanglin Yang, Jiaying Lin, Xiaobing Liu

**Affiliations:** ^1^Department of Vascular Surgery, Shanghai Ninth People’s Hospital, Shanghai Jiao Tong University School of Medicine, Shanghai, China; ^2^Department of Assisted Reproduction, Shanghai Ninth People’s Hospital, Shanghai Jiao Tong University School of Medicine, Shanghai, China; ^3^Department of Vascular Surgery, Fengcheng Hospital Affiliated to Shanghai Ninth People’s Hospital, Shanghai Jiao Tong University School of Medicine, Shanghai, China

**Keywords:** CuBiS_2_ nanoparticles, computed tomography, photothermal therapy, arterial restenosis, endovascular treatment

## Abstract

Chronic inflammation mediated by artery infiltrated macrophages plays critical role in artery restenosis after endovascular therapy. Evidence has demonstrated the potential ability of photothermal therapy (PTT) in eliminating chronic inflammation by targeting inflammatory cells including macrophages. Recently, increasing attention has been payed to copper chalcogenide nanocrystals doped of radiocontrast agent, e.g., bismuth (Bi) for computed tomography (CT) guided PTT. However, the application of imaging guided PTT in preventing artery restenosis is lacking and limited. Herein, a novel multifunctional CuBiS_2_ nanoparticles (CuBiS_2_ NPs) were synthesized for CT imaging guided PTT in artery re-stenosis prevention. The optimum amount and other conditions of CuBiS_2_ NPs were optimized to exert the maximum ablation effect on macrophages with good biocompatibility. *In vivo* carotid injury model revealed that CuBiS_2_ NPs exhibited promising therapeutic effect on inhibition of artery stenosis by eliminating macrophages with excellent CT imaging ability. The recent study highlights a new cost-effective metal nanostructures-based nanotechnology in prevention of artery restenosis after endovascular therapy.

## Introduction

Atherosclerosis remains a devastating disease affecting cardio/cerebrovascular system with high morbidity and mortality (Toyohara et al., 2020). Although endovascular treatment i.e., percutaneous transluminal angioplasty (PTA) and stent implantation is somehow considered the treatment of choice when dealing with atherosclerotic stenosis or occlusion (Baumgartner et al., 2018), it would unavoidably lead to the injury of arterial endothelium, release of various cytokines and chemokines, and subsequent recruitment of circulating monocytes to the arterial wall (Koelwyn et al., 2018; Morley et al., 2018). Once activated, infiltrated monocytes differentiate into macrophages, mediating chronic inflammation of the injured artery (Koelwyn et al., 2018). Evidence has been growing that inflammatory macrophages play crucial role in artery restenosis after endovascular treatment (Sun et al., 2016; Pei et al., 2019). Infiltrated macrophages secrete matrix metalloproteinase (MMP) to degrade extracellular matrix and pro-inflammatory cytokines to stimulate the proliferation and transformation of smooth muscle cell (SMC) via different signaling pathways, eventually leading to the negative remodeling of the artery, i.e., artery restenosis (Yamashita et al., 2015; Zhao et al., 2017). Therefore, depletion of local aggregated inflammatory macrophages would provide a powerful mean to prevent artery restenosis.

Due to excellent properties of tissue penetration and minimally invasive, near-infrared (NIR) light driven photothermal therapy (PTT) based on nanomaterials could induce apoptosis and cell death by targeting intracellular protein and DNA (Al-Barram, 2020; Zhang et al., 2020; Zhi et al., 2020). With the advantages above and novel nanoparticles being developed (such as modified/decorated metal-organic framework based nanomaterial), nanotech PTT has been shown to be greatly applied in the anti-tumor theranostics, cardiovascular diseases and nervous system diseases, etc. (Shan et al., 2018; Luo et al., 2019; Wang et al., 2019; Pan et al., 2020; Zhou et al., 2020). Using ZD2-engineered gold nanostar@ metal-oragnic framework nanoprobes, it has been reported that the nanoprobes based PTT was efficient in treating triple-negative breast cancer with good magnetic resonance imaging property (Zhang et al., 2018). Nevertheless, we have previously reported that other nanoparticles, e.g., CuCo_2_S_4_ nanocrystals, MoO_2_ nanoclusters, Fe_3_S_4_ nanoparticles, polypyrrole nanoparticles, and gold nanorods as PTT agents, can effectively alleviate inflammation by eliminating inflammatory macrophages (Peng et al., 2015; Qin et al., 2015; Wang et al., 2019; Zhang et al., 2019). Collectively, growing evidence suggest that nanoparticles as PTT agents may be an attractive and promising therapeutic target for chronic inflammation, including artery re-stenosis. However, the potential use of nanoparticle-based PTT in preventing artery restenosis after endovascular intervention is still lacking.

Metal sulfides are kinds of ideal candidate materials for photothermal applications, but their band gaps are usually too large to absorb significant fractions of NIR light and it has been gathering interest that by means of combining Cu^+^ and Bi^3+^, the ternary sulfides CuBiS_2_ is formed (Dufton et al., 2012). CuBiS_2_ is a semiconductor material with an energy gap distributed between 1.5 and 2.1 eV with good light absorption capability. Askari and Askari (2019) reported that CuBiS_2_ demonstrated good photothermal property and showed anticancer effect on AGS cancer cell line via apoptosis pathway *in vitro*. On one hand, due to non-carcinogenic or toxic properties, bismuth is considered safe in pharmaceutical application and some of its components or compounds as an anti-inflammatory, antiviral and antifungal agents are widely used in clinical treatment (Tiekink, 2002; Iuchi et al., 2008). Moreover, accumulating evidence has been reported that by doping of radiocontrast agent, e.g., bismuth, copper chalcogenide nanocrystals exhibited excellent property for CT guided PTT, which has attracted great attention (Liu et al., 2015). However, evidence of CuBiS_2_ based PTT in preventing artery re-stenosis is lacking.

In current study, CuBiS_2_ nanoparticles (CuBiS_2_ NPs) were synthesized and characterized *in vitro*/*vivo* and photothermal properties of the nanoparticles were further evaluated. Moreover, the cytotoxicity and PTT effect of CuBiS_2_ NPs on inflammatory macrophages were also assessed *in vitro*. Carotid inflammation/endothelium injury model was established by using 29G Syringe needle mimicking endothelia damage after endovascular intervention. After that, PTT was conducted based on local injection of CuBiS_2_ NPs to the surrounding of the injured carotid artery. Of note, we specially evaluated the imaging ability of CuBiS_2_ NPs for *in vivo* tracking by small animal CT device. Histologic analysis demonstrated the effect of PTT in attenuating artery wall inflammation and stenosis. Also, histologic analysis of major organs and blood examination were performed to evaluate the biocompatibility of CuBiS_2_ NPs.

## Materials and Methods

### Materials

Raw264.7 (mouse macrophage) was obtained from Fuheng Cell Bank, Fudan University (Shanghai, China) for the *in vitro* study. High glucose (4,500 mg^–1^ mL) Dulbecco′s Modified Eagle′s Medium (DMEM), penicillin/streptomycin and fetal bovine serum were purchased from Gibco (Carlsbad, CA, United States). The CD68 antibody and corresponding 2nd antibody were purchased from Thermo Fisher Scientific (United States). Cell Counting Kit-8 (CCK-8) and Calcein-AM/PI Double Stain Kit were purchased from Thermo Fisher Scientific (United States).

### CuBiS_2_ NPs Synthesis and Characterization

One-step hydrothermal process (Wang et al., 2019) was used for the synthesis of the CuBiS_2_ NPs according to the method described previously. Briefly, 0.256 g CuCl_2_.2H_2_O and 0.16 g BiCl_3_ was mixed and stirred with 30 mL anhydrous alcohol and 50 mL glycerol by magnetic stirrer, during which thiourea resolution (i.e., 0.19 g thiourea dissolved in 10 mL anhydrous alcohol) was dropped and stirred together for 10 min. After that, the mixture solution was transferred to a flat flask for constant temperature water bath heating at 60°C for 1 h and moved to a Teflon lined autoclave to be heated at 160°C for 12 h. After centrifugation (5,000 r/min), the precipitate was then collected and washed with 75% ethanol and deionized water three times.

Scanning electron microscopy (SEM) characterized the size and morphology properties of the CuBiS_2_ NPs. UV-vis absorption spectra were measured by Jasco V-7000 UV-visible-NIR spectrophotometer (Tokyo, Japan). X-ray diffractometer (XRD) analysis were conducted using Aeris X-ray diffractometer (Malvern, United Kingdom). Fourier transform infrared (FTIR) spectra were analyzed by KBr pellet methods using TruDefender^TM^ FTX/FTXi infrared spectrometer (Thermo Fisher Scientific; United States).

The power of 808 nm semiconductor laser (Hangzhou Qiulai Optoelectronics Technology Co. Ltd., China) could be externally adjusted (average 1.5 W). Calibration of the output power of lasers was conducted by using a hand-held optical power meter (Newport model 1918-C, CA, United States).

### Cell Culture and Characterization

Raw264.7 macrophages were cultured in DMEM medium (4,500 mg^–1^ mL glucose, with 10% FBS and 1% streptomycin/penicillin) and maintained at 37°C in a humidified 5% CO_2_ atmosphere. Cellular immunofluorescence (IF) staining was performed to identify Raw264.7 macrophage properties by the fluorescence microscope (Olympus, Japan).

### Cytotoxicity and Cell Viability

The cytotoxicity of CuBiS_2_ NPs on macrophages was evaluated in the absence of PTT. Raw264.7 was co-cultured with CuBiS_2_ NPs at different concentrations (0, 80, 160, 320 mg/mL) for 12 h. CCK-8 cell proliferation assay was used to measure the cell viability of macrophages after co-incubation with CuBiS_2_ NPs, following which the safe concentration of CuBiS_2_ NPs was determined and utilized for the subsequent *in vitro* or *in vivo* experiments. Next, the photothermal effects of CuBiS_2_ NPs on macrophages was assessed. Raw264.7 was co-cultured with CuBiS_2_ NPs at a predetermined concentration above for 12 h and subjected to 808 nm NIR laser irradiation for 5 min. After that, the irradiated macrophages stained with Calcein AM/PI (Calcein AM labeled-green, PI labeled-red, respectively) were observed under fluorescence microscope to discriminate living and dead cells. Flow cytometry (FCM) was performed to assess cell apoptosis analysis after Annexin V/PI staining following the procedures described previously (Wang et al., 2019).

### Intracellular SEM

CuBiS_2_ NPs engulfed by macrophages was observed by intracellular Transmission Electron Microscope (TEM; JEM-1400, Tokyo, Japan).

### Animal Model and Photothermal Therapy

The animal experiment protocol was approved by Ethics Review Committee of Shanghai Ninth People’s Hospital, Shanghai Jiao Tong University School of Medicine. Male, 8-week-old apolipoprotein E knockout mice (ApoE^–/–^ mice; Shanghai Southern Model Biological Co., Ltd.) were raised under specific pathogen-free (SPF) conditions. The method for artery inflammation and endothelium injury mice model is briefly described as follows: First anesthetize the ApoE^–/–^ mouse by intraperitoneal injection of pentobarbital sodium (40 mg/kg), after that the left common carotid artery (CCA) was dissected, and blocked by a blocking clamp at the proximal end, an incision was then made at the distal end of CCA, following which a 29G needle (BD Insulin Syringe Ultra-Fine^®^) was inserted to the CCA. To mimic the endothelium injury by endovascular treatment, the needle in CCA was rotated for three circles and pushed forward-back for three times. After closing the incision, the carotid artery was sheathed with a constrictive silica collar as previously descripted (Wang et al., 2019).

After 14 days, 100 mL (160 mg/mL) CuBiS_2_ NPs were injected into the left carotid arteries, while the contralateral right carotid artery was sham-operated to serve as intra-animal control (without silica collar). Twelve hours after injection, all the necks were irradiated at 808 nm NIR laser with power density of 0.5 W cm^–2^ for 5 min. GX-300 photothermal medical device was used to record the temperature of full-body infrared thermal images dynamically.

### Computed Tomography *in vivo*

Small animal CT scanning was used to determine the imaging ability of CuBiS_2_ NPs. Endothelium injury model mice received CT scanning before and after perivascular injection with the CuBiS_2_ NPs (160 mg/mL, 100 mL, per mouse). CT data were acquired using X-ray voltage biased to 50 kVp with a 670 μA anode current with projection angles of 720°. Afterward, 3-dimensional CT imaging was established to observe the distribution of CuBiS_2_ NPs around the left carotid artery.

### Tissue Histological Findings and Blood Examination

Fourteenth day after PTT, all the mice were sacrificed for histopathological examination. Both sham-operated and collared carotid arteries were harvested. The tissue infiltrating macrophages was stained for its surface maker CD68 and the immunofluorescent signal was detected by microscope which was further quantified using Image-Pro Plus software. The number of infiltrated macrophages were counted by two investigators blinded to group information. Hematoxylin-eosin (HE) staining was performed to determine the thickness of the intima-media of arteries by the Image-pro Plus software.

To assess the biocompatibility and toxicity of the CuBiS_2_ NPs *in vivo*, major viscera, e.g., heart, liver, spleen, lung and kidney were made at 4–6 nm sections slides for HE staining. Five age-sex matched healthy ApoE^–/–^ mice were sacrificed as control. Biochemical parameters of the blood samples were measured in Shanghai Research Center.

### Statistics Analysis

Quantitative data was represented as means ± standard deviation (SD), and one-way *Annona* analysis was used to compare the difference among multiple groups. Student’s *t*-test was used when appropriate. A *P*-value < 0.05 was considered statistically significant. All data are representative of at least three independent experiments.

## Results and Discussion

### Characterization of CuBiS_2_ NPs

**FIGURE 1:**
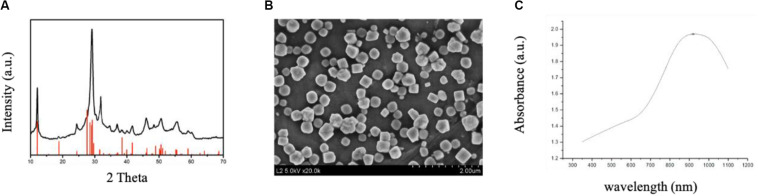
Characterization of CuBiS_2_ NPs. **(A)** XRD pattern and the corresponding standard pattern of CuBiS_2_ NPs. **(B)** SEM images of CuBiS_2_ NPs, Scale bars: 2 μm; **(C)** UV-vis-NIR absorption spectrum of CuBiS_2_ NPs.

CuBiS_2_ NPs were synthesized according to one-step hydrothermal method described previously. Figure 1A shows the XRD pattern of the as-synthesized products. The pattern of the sample can be matched well with the emplectite CuBiS_2_ phase (JCPDS no. 43-1473), without no other peaks. EDS analysis (Supplementary Figure S1) showed that the products was composed of three elements (i.e., Cu, Bi, and S), further indicating the high purity of the CuBiS_2_ NPs. As shown in Figure 1B, SEM results confirmed the products were nanoparticles with an average diameter of 240 nm (range from 180 to 400 nm). Figure 1C exhibits the UV-vis absorbance spectrum of the aqueous dispersion of CuBiS_2_ NPs. It showed an intense absorption band centered at 910 nm. The strong NIR absorption made the nanoparticles possess the potential of to be PTT agents. Moreover, the zeta potential of CuBiS_2_ NPs (160 mg/mL) was recorded by zeta potential analyzer (Nicomp Z3000) for 5 min, the result of which showed that the average zeta potential value was -1.86 mV.

**FIGURE 2:**
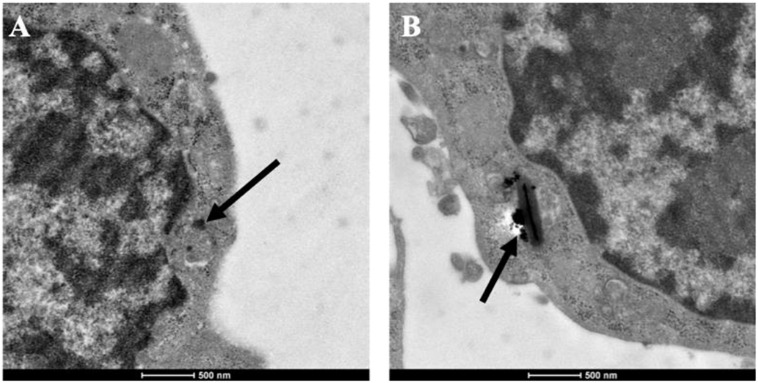
Representative of TEM images of Raw264.7 co-incubated with CuBiS_2_ NPs. Scale bar: 500 nm. **(A,B)** Macrophages incubated with 160 mg/mL CuBiS_2_ NPs for 6 and 12 h, respectively. Scale Bar: 500 nm.

As an important member of mononuclear phagocytic system, macrophages have powerful phagocytosis. Therefore, before determining the cytotoxicity and photothermal properties of CuBiS_2_ NPs on Raw 264.7, we explored the phagocytosis of macrophages toward the CuBiS_2_ NPs by TEM. The results showed efficient phagocytosis of CuBiS_2_ NPs with no obvious accumulation or sever damage to other organelles of macrophages (Figure 2).

**FIGURE 3:**
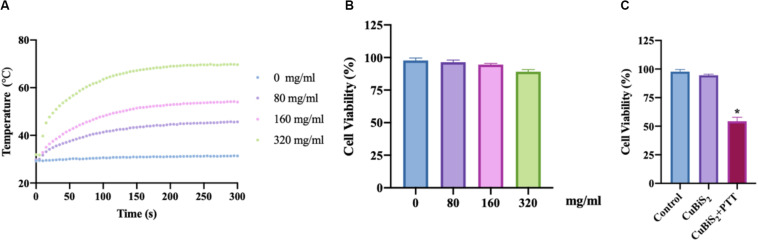
Screening the optimal concentration of CuBiS_2_ NPs for photothermal therapy. **(A)** *In vitro* photothermal effect of PBS and CuBiS_2_ NPs in aqueous solution at different concentrations. **(B)** Relative cell viabilities of macrophages after co-incubation with CuBiS_2_ NPs at different concentrations for 12 h without photothermal therapy. **(C)** Relative cell viabilities of macrophages after co-incubation with CuBiS_2_ NPs at different concentrations for 12 h under photothermal therapy.

To evaluate the photothermal effect of CuBiS_2_ NPs, the temperature evolution at different CuBiS_2_ NPs concentration (0, 80, 160, 320 mg/mL) under continuous 808 nm wavelength laser irradiation for 300 s were recorded, showing that the temperature was elevated in dramatic and smooth pattern with increasement of CuBiS_2_ NPs concentration (Figure 3A). Cytotoxicity of CuBiS_2_ NPs on macrophages should be considered before other biomedical applications. To this end, Raw264.7 was co-cultured with CuBiS_2_ NPs for 12 h in the absence of PTT. Subsequently, the CCK-8 assay was conducted to measure the concentration-dependent cytotoxic effect of CuBiS_2_ NPs on Raw264.7 (Figure 3B). Finally, no significant difference on cell cytotoxicity was identified between the CuBiS_2_ NPs group and the control group below the concentration of 160 mg/mL, while significant cytotoxicity was observed when macrophages were co-incubated with CuBiS_2_ NPs at concentration above 160 mg/mL, indicating that CuBiS_2_ NPs exhibited good biocompatibility at concentration of 160 mg/mL (Figure 3B).

**FIGURE 4:**
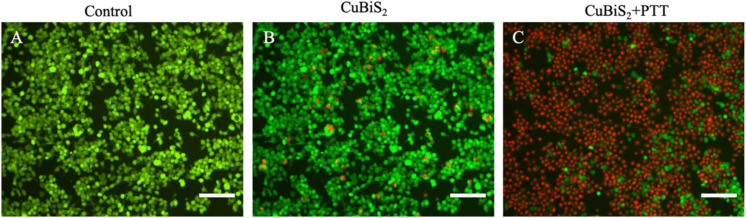
Photothermal effect of CuBiS_2_ NPs on macrophages by Calcein AM/PI and flow cytometry. **(A–C)** Representative images of live (green) or dead (red) cells without or with CuBiS_2_ NPs co-incubation (160 mg/mL), respectively. Scale Bar: 100 μm.

To investigate the applicability of CuBiS_2_ NPs based PTT, *in vitro* evaluation of their photothermal efficacy was performed. Raw264.7 macrophages were co-cultured with CuBiS_2_ NPs at 160 mg/mL for 12 h, and then subjected to 808 nm NIR irradiation (0.3 W/cm^2^). Calcein AM/PI results demonstrated that few dead cells were observed in the control group, and CuBiS_2_ NPs group (Figures 4A,B). While about 60% dead cells were observed in the CuBiS_2_ NPs + PTT group (Figure 4C). In accordance with Calcein AM/PI, the CCK-8 assay showed similar results (Figure 3C). The above results showed that CuBiS_2_ NPs based PTT exhibited excellent photothermal property with remarkable macrophages death being observed.

**FIGURE 5:**
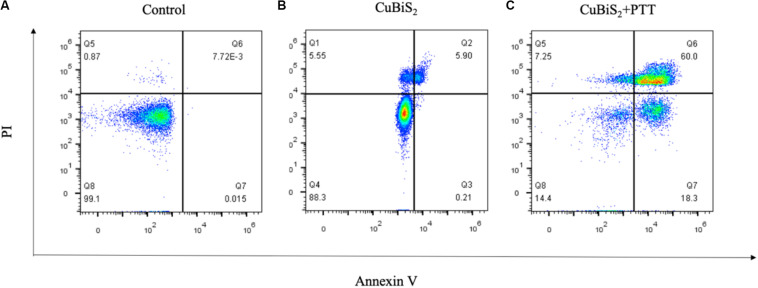
CuBiS_2_ NPs based PTT induced apoptosis of macrophages. Flow cytometry analysis of Raw264.7 apoptosis after different treatments: **(A)** Control; **(B)** CuBiS_2_; **(C)** CuBiS_2_ + PTT. Cell apoptosis was detected by flow cytometry with annexin-V-FITC and PI. CuBiS_2_ NPs + PTT group showed higher early (Q7) and mid/late (Q6) apoptosis index than CuBiS_2_ group.

Apoptosis is an important type of programmed cell death which can be induced by thermal effect, whether it is involved in CuBiS_2_ NPs based PTT induced cell death is unknown. Annexin V/PI duo-stanning detects the signals of Annexin V and the impermeable nucleic acid dye, respectively, which can quantify the proportion of mid- and late-apoptotic cells, we used Annexin V/PI stanning to determine the CuBiS_2_ NPs based PTT effect on macrophages and discriminate cell apoptosis and necrosis. Toward this end, FCM was performed showing that compared with CuBiS_2_ NPs group, CuBiS_2_ NPs + PTT group exhibited a significantly higher either early apoptosis (18.3 vs. 0.21%) or mid/late apoptosis (60 vs. 5.9%) index of macrophages with statistical difference (Figure 5). In contrast to programmed cell death, necrosis is the death process in which cells are subject to strong physical and chemical or biological factors that cause disordered changes in cells. As shown in Figure 5, the necrosis index of macrophages was identical between CuBiS_2_ NPs group and CuBiS_2_ NPs + PTT group, indicating that the type of CuBiS_2_ NPs based PTT induced macrophages death was mainly apoptosis instead of necrosis. The above results demonstrated that CuBiS_2_ NPs based PTT could ablate macrophages effectively by inducing cell apoptosis (Figures 5A–C), which possesses great potential to alleviate chronic inflammation mediated by macrophages.

### CT Imaging Assessment and Photothermal Effect of CuBiS_2_ NPs *in vivo*

**FIGURE 6:**
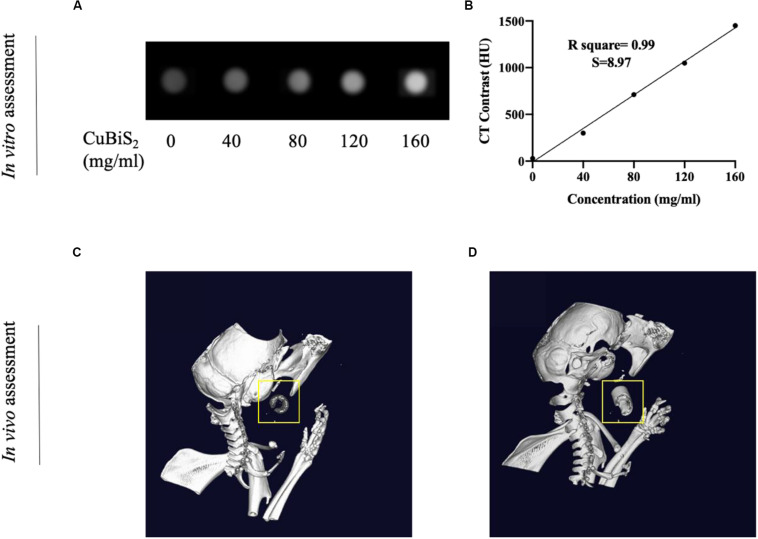
Demonstration of computed tomography imaging property of CuBiS_2_ *in vitro and in vivo*. **(A)** The CT signals intensity of different CuBiS_2_ concentration *in vitro*. **(B)** CT value (HU) of the CuBiS_2_ NPs as a function of the concentration of NPs. **(C,D)** 3-D CT imaging of mice without and with CuBiS_2_ NPs being injected to the surrounding of the carotid artery, respectively.

Nanomaterials have drawn great attention over the decades and been widely utilized in biomedicine (Cole et al., 2015; Zhang et al., 2016). Nanoplatforms can integrate imaging moieties and therapeutic species flexibly, which has been widely applied in cancer diagnosis and treatment (Liu et al., 2007). Among numbers of investigations, CT imaging guided PTT has been widely reported and highly recognized. Conventional contrast agents have the disadvantages of short imaging time and potential nephrotoxicity, while new nanocrystal contrast agents such as Bi_2_S_3_, TaOx, and other nanoparticles (NPs) overcome the above shortcomings and has high absorption coefficient (Ai et al., 2011; Lee et al., 2012; Leeuwenburgh et al., 2013; Cheng et al., 2014). With high density (ρ) and atomic number (Z), Bi element was reported to possess high attenuation coefficient of X-ray (Elsabahy et al., 2015). With long vascular half-life, Bi_2_S_3_ nanoplates was reported to gain considerable potential to achieve enhanced CT efficacy with lower agent dose in future clinical use (Rabin et al., 2006). In addition to the photothermal ablation ability, CT imaging property of CuBiS_2_ NPs was further assessed *in intro* and *in vivo* on the bases of pre-determined concentration for cytotoxicity and cell viability evaluation *in vitro*. Figure 6 presents the CT image of aqueous dispersions of the CuBiS_2_ NPs with different concentration, showing that CT signal intensity enhanced with increased concentrations of the CuBiS_2_ NPs. Meanwhile, the Hounsfield units (HU) values increased linearly with the concentration of the CuBiS_2_ NPs. As shown in Figures 6A,B, CuBiS_2_ NPs at concentration of 160 mg/ml exhibited excellent *in vitro* imaging property, which was further tested *in vivo* showing that distinct difference was observed in CT images of CuBiS_2_ NPs surrounding inflammatory artery, demonstrating good CT imaging property of CuBiS_2_ NPs (Figures 6C,D).

**FIGURE 7:**
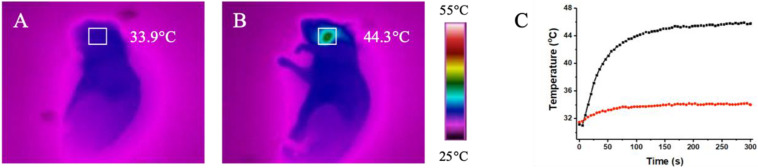
*In vivo* thermal effect of CuBiS_2_ NPs *in vivo*. **(A,B)** Representative of infrared thermal images of the mice after being injected with PBS or CuBiS_2_ NPs under laser irradiation. **(C)** Temperature changes according to irradiation time in corresponding squared regions (red: PBS control; black: CuBiS_2_ NPs group).

Endovascular repairs would unavoidably cause mechanical injury to endothelial cells and activates the endothelium to express massive adhesive molecules and chemokines, which promotes the recruitment of monocytes, accelerates differentiation of monocytes into macrophages and aggravate artery inflammation, leading the restenosis after endovascular treatment (Sun et al., 2016; Pei et al., 2019). According to above results, we speculate that the macrophages infiltrated in the injured lesion of artery endothelium engulf locally injected non-toxic CuBiS_2_ NPs and provide opportunities for *in vivo* non-invasive PTT to attenuate arterial inflammation and stenosis. Toward this end, a carotid artery inflammation/endothelium injury model was conducted in ApoE^–/–^ mice by mechanical injury to intima using a 29G Syringe needle mimicking endovascular treatment related endothelium injury to further validate the feasibility of CuBiS_2_ NPs based PTT for inhibiting artery restenosis (Supplementary Figure S2). The mice received local injection of a solution of the CuBiS_2_ NPs (160 mg/mL, 100 mL) around the left carotid artery and subjected to NIR laser irradiation (808 nm, 0.5 W cm^–2^) 12 h. An equivalent volume of PBS was injected as control. The local temperatures of the left necks were recorded by an infrared thermal camera dynamically. In mice injected with CuBiS_2_ NPs, the local surface temperature of the left neck gradually increased to 45°C rapidly within 5 min, while non-dramatic increase of the local surface temperature was recorded in the control group being injected PBS (Figure 7). After 14 days, the mice were sacrificed, and the carotid arteries were collected for HE staining and immunofluorescence examination.

**FIGURE 8:**
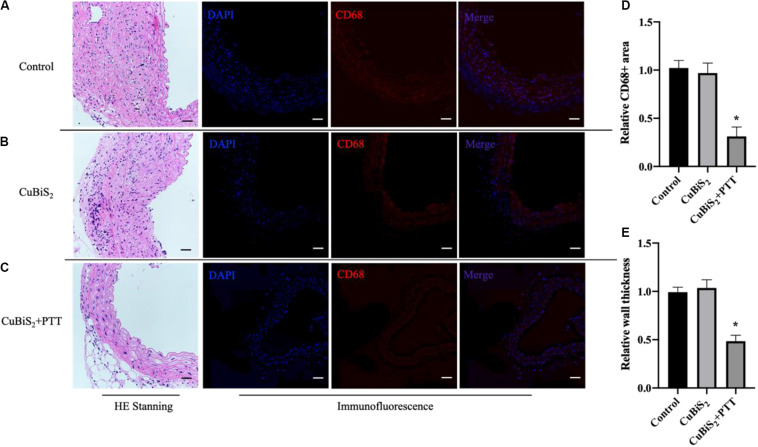
CuBiS_2_ NPs based PTT mitigated arterial inflammation *in vivo*. **(A–C)** Representative of HE and Immunofluorescence stanning of carotid artery from control group, CuBiS_2_ group and CuBiS_2_ + PTT group, respectively. **(D,E)** Representative of CD68 + macrophages positive area and artery wall thickness in each group, respectively. Scale bar: 50 μm.

The effectiveness of CuBiS_2_ NPs based PTT for alleviating arterial inflammation was further assessed by IF (Figures 8A–D), the results of which showed that the artery-infiltrating macrophages in the CuBiS_2_ NPs + PTT group reduced greatly. The HE staining exhibited that the thickness of arterial intima-media in the CuBiS_2_ NPs + PTT group was much thinner than that of the CuBiS_2_ NPs group and the control group (Figure 8E). However, there was no significant difference between the control group and the CuBiS_2_ NPs group. Thus, the results suggested CuBiS_2_ NPs as photothermal agents and 808 nm NIR PTT can effectively eliminate inflammatory macrophages infiltrated in the layer of artery to inhibit arterial inflammation and stenosis.

### Biocompatibility of the CuBiS_2_ Nanoparticles

**FIGURE 9:**
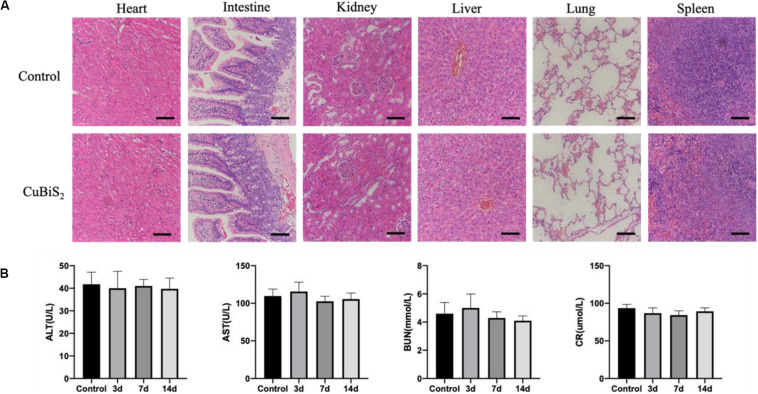
*In vivo* biocompatibility test of CuBiS_2_ NPs. **(A)** HE staining of major viscera showed no obvious histopathological changes in heart, intestine, kidney, liver, lung, or spleen between the control group and the CuBiS_2_ group. Scale bar: 50 μm. **(B)** Laboratory blood test of control group and CuBiS_2_ group at different time points (3, 7, 14 days) after CuBiS_2_ NPs injection, including alanine aminotransferase (ALT), aspartate aminotransferase (AST), blood urea nitrogen (BUN), and creatinine (CR).

Of note, favorable biocompatibility of NPs should be considered and guaranteed for living body. Mice from CuBiS_2_ NPs group and control group (*n* = 4, each) were sacrificed 14 days after PTT. Paraffin-embedded sections (4 nm) of major organs were stained with hematoxylin-eosin (HE) dye. No obvious histopathological changes were found between the two groups (Figure 9A). No obvious cell degeneration and necrosis were observed in major viscera (Figure 9A). Blood samples were tested using Elisa method. Statistical difference was not gained between the two groups in terms of alanine aminotransferase (ALT), asparate aminotransferase (AST), blood urea nitrogen (BUN), or creatinine (Cr) (Figure 9B). As dysfunction of endothelial cells contributed to negative remodeling the artery (e.g., artery restenosis), Evan’s Blue stanning of the carotid artery from each group was performed to test the potential side-effect of CuBiS_2_ NPs on reendothelialization, showing that no difference was found between the groups (Supplementary Figure S3). These results show that CuBiS_2_ NPs as PTT agents have no toxic effects on the major organ function, and thus are safe for use *in vivo*.

## Conclusion

In summary, CuBiS_2_ NPs were successfully synthesized by one-step hydrothermal method. The NPs showed intense NIR absorption due to the defect structure, thus were demonstrated excellent photothermal performance. Due to the High X-ray attenuation coefficient of bismuth, CuBiS_2_ NPs possessed CT imaging ability. CuBiS_2_ NPs based PTT could eliminate macrophages *in vitro* and *in vivo*, expanding the understanding of CuBiS_2_ NPs as efficient PTT agents for arterial inflammation and restenosis after endovascular treatment.

## Data Availability Statement

All datasets generated for this study are included in the article/Supplementary Material.

## Ethics Statement

The animal study was reviewed and approved by the Shanghai Ninth People’s Hospital, Shanghai Jiao Tong University School of Medicine.

## Author Contributions

XW and XL contributed to the model development, code development, data generation and analysis, writing, and editing the manuscript. JL and GY contributed to the model development, data analysis, and editing the manuscript. KL and RW contributed to the model development, data analysis, writing, and editing the manuscript. All the authors contributed to discussion of the results.

## Conflict of Interest

The authors declare that the research was conducted in the absence of any commercial or financial relationships that could be construed as a potential conflict of interest.
