# Supplementary material for: Multifunctional CuBiS2 Nanoparticles for Computed Tomography Guided Photothermal Therapy in Preventing Arterial Restenosis After Endovascular Treatment
Source: Front Bioeng Biotechnol. 2020 Oct 21;8:585631. doi: 10.3389/fbioe.2020.585631 (PMC7609917; doi:10.3389/fbioe.2020.585631)
Supplement: Supplementary file 1 [file Data_Sheet_1.docx]

**Supporting Information**

**Multifunctional CuBiS_2_ nanoparticles for computed tomography guided photothermal therapy in preventing arterial restenosis after endovascular treatment**

Xiaoyu Wu^1*^, Kun Liu^1^, Ruihua Wang^1^, Guanglin Yang^1^^*^, Jiaying Lin^2^*, Xiaobing Liu^1,3^*

^1^Department of Vascular Surgery, Shanghai Ninth People’s Hospital, Shanghai Jiao Tong University School of Medicine, Shanghai 200011, China.

^2^Department of Assisted Reproduction, Shanghai Ninth People’s Hospital, Shanghai Jiao Tong University School of Medicine, Shanghai 200011, China.

^3^Department of Vascular Surgery, Fengcheng Hospital affiliated to Shanghai Ninth People’s Hospital, Shanghai Jiao Tong University School of Medicine, Shanghai 201411, China.

*Correspondence:

Xiaoyu Wu, Email: wxy_cmu@aliyun.com; Guanglin Yang, Email: yangglbs@hotmail.com; Jiaying Lin, Email: 13818761532@126.com; Xiaobing Liu, Email: benny_liuxb@163.com.


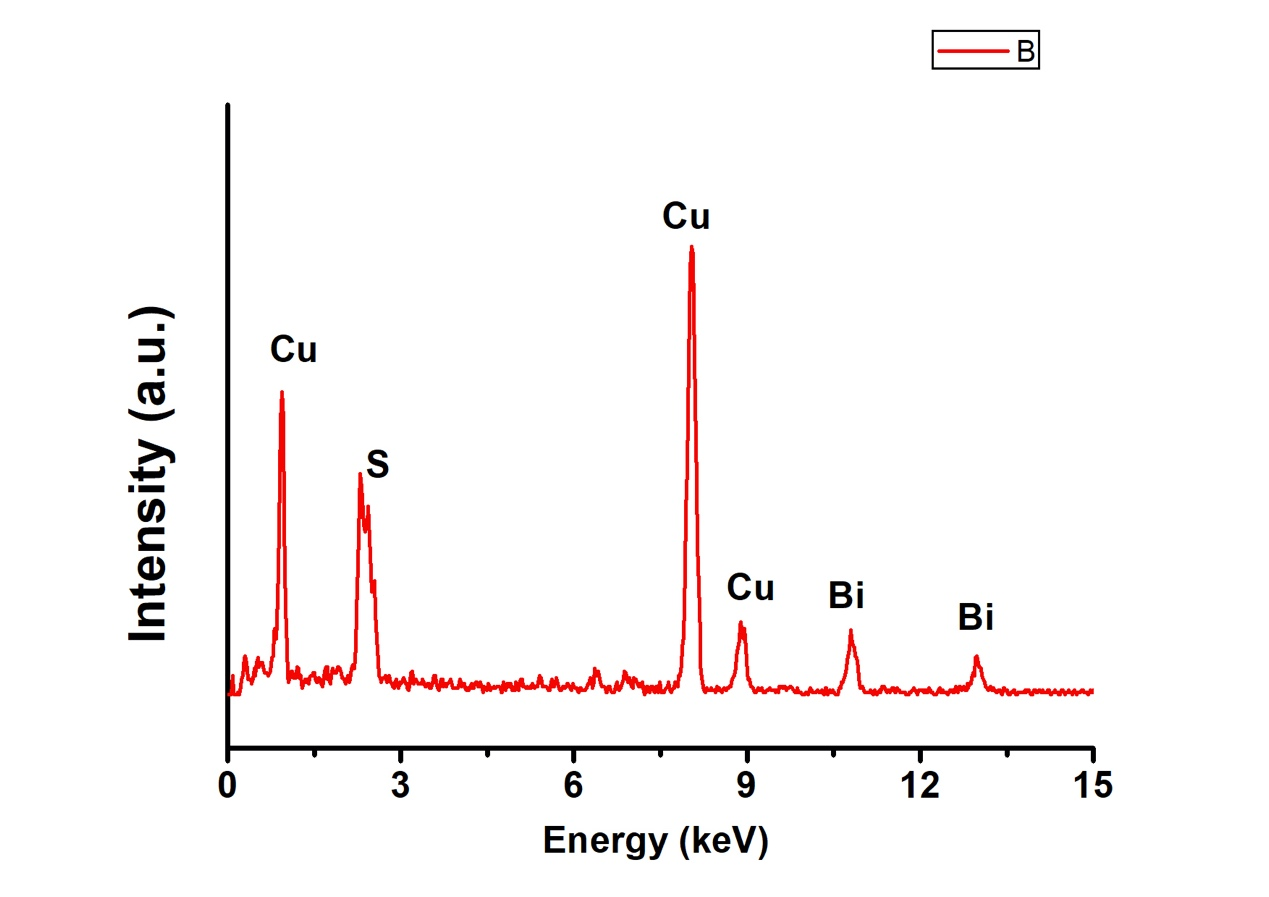


Figure S1. EDS analysis of CuBiS_2_ NPs.


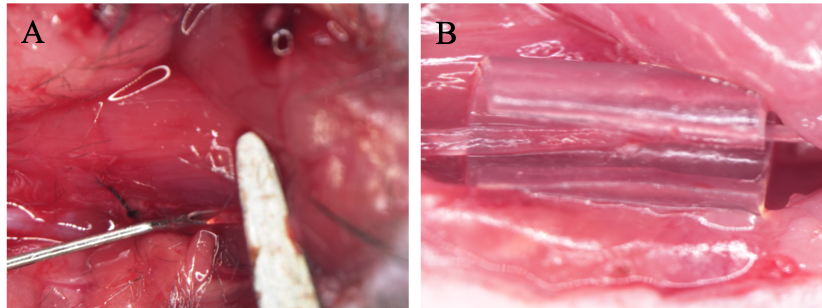


Figure S2. Artery inflammation/endothelium injury model was established by using a 29G needle (BD Insulin Syringe Ultra-Fine^®^ and silica collar. A: Carotid artery was dissected, and endothelium was injured by a needle. B: Carotid artery was covered by a silica collar.


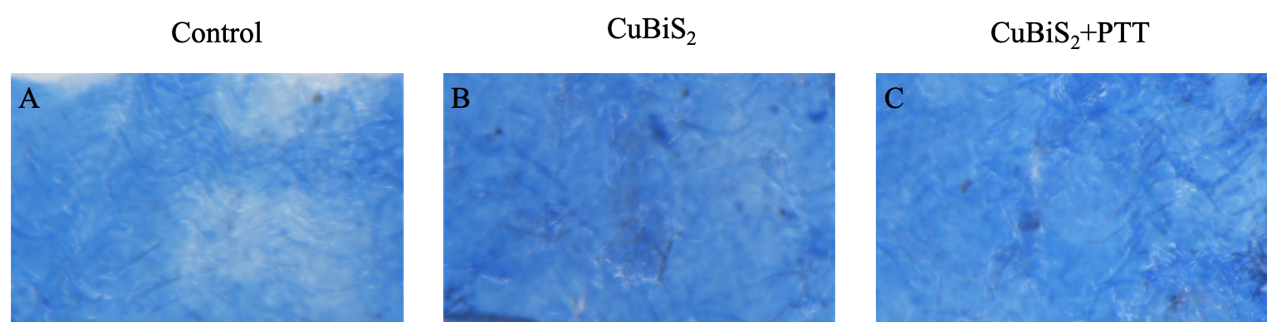


Figure S3. Reendothelialization of the damaged intima was detected by Evan’s blue stanning. A-C: represented the reendothelialization of the damaged intima in control group, CuBiS2 group and CuBiS2 +PTT group respectively.
